# ^18^F-Sodium fluoride PET/CT predicts overall survival in patients with advanced genitourinary malignancies treated with cabozantinib and nivolumab with or without ipilimumab

**DOI:** 10.1007/s00259-019-04483-5

**Published:** 2019-09-14

**Authors:** Ilhan Lim, Maria Liza Lindenberg, Esther Mena, Nicholas Verdini, Joanna H. Shih, Christian Mayfield, Ryan Thompson, Jeffrey Lin, Andy Vega, Marissa Mallek, Jacqueline Cadena, Carlos Diaz, Amir Mortazavi, Michael Knopp, Chadwick Wright, Mark Stein, Sumanta Pal, Peter L. Choyke, Andrea B. Apolo

**Affiliations:** 1 Molecular Imaging Program, Center for Cancer Research, National Cancer Institute, National Institutes of Health, 10 Center Drive, B3B403, Bethesda, MD 20892 USA; 2 Department of Nuclear Medicine, Korea Cancer Center Hospital, Korea Institute of Radiological and Medical Sciences (KIRAMS), Seoul, South Korea; 3 Genitourinary Malignancies Branch, Center for Cancer Research, National Cancer Institute, National Institutes of Health, Bethesda, MD USA; 4 Biometric Research Branch, Division of Cancer Treatment and Diagnosis, National Cancer Institute, National Institutes of Health, Bethesda, MD USA; 5 Division of Medical Oncology, Department of Internal Medicine, The Ohio State University Comprehensive Cancer Center, Columbus, OH USA; 6 Wright Center of Innovation in Biomedical Imaging, Division of Imaging Science, Department of Radiology, The Ohio State University Wexner Medical Center, Columbus, OH USA; 7 Wright Center of Innovation in Biomedical Imaging, Division of Nuclear Medicine and Molecular Imaging, Department of Radiology, The Ohio State University Wexner Medical Center, Columbus, OH USA; 8 Division of Genitourinary Medical Oncology, Department of Medicine, Rutgers Cancer Institute of New Jersey and Rutgers Robert Wood Johnson Medical School, New Brunswick, NJ USA; 9 City of Hope Comprehensive Cancer Center, Duarte, CA USA; 10 Center for Cancer Research, National Cancer Institute, National Institutes of Health, 10 Center Dr., 13N240, MSC 1906, Bethesda, MD 20892 USA

**Keywords:** Fluoride PET/CT, NaF PET/CT, Immune checkpoint inhibitor, Cabozantinib, Genitourinary malignancy

## Abstract

**Purpose:**

We evaluated the prognostic value of ^18^F-sodium fluoride (NaF) PET/CT in patients with urological malignancies treated with cabozantinib and nivolumab with or without ipilimumab.

**Methods:**

We prospectively recruited patients with advanced urological malignancies into a phase I trial of cabozantinib plus nivolumab with or without ipilimumab. NaF PET/CT scans were performed pre- and 8 weeks post-treatment. We measured the total volume of fluoride avid bone (FTV) using a standardized uptake value (SUV) threshold of 10. We used Kaplan-Meier analysis to predict the overall survival (OS) of patients in terms of SUVmax, FTV, total lesion fluoride (TLF) uptake at baseline and 8 weeks post-treatment, and percent change in FTV and TLF.

**Result:**

Of 111 patients who underwent NaF PET/CT, 30 had bone metastases at baseline. Four of the 30 patients survived for the duration of the study period. OS ranged from 0.23 to 34 months (m) (median 6.0 m). The baseline FTV of all 30 patients ranged from 9.6 to 1570 ml (median 439 ml). The FTV 8 weeks post-treatment was 56–6296 ml (median 448 ml) from 19 available patients. Patients with higher TLF at baseline had shorter OS than patients with lower TLF (3.4 vs 14 m; *p* = 0.022). Patients with higher SUVmax at follow-up had shorter OS than patients with lower SUVmax (5.6 vs 24 m; *p* = 0.010). However, FTV and TLF 8 weeks post-treatment did not show a significant difference between groups (5.6 vs 17 m; *p* = 0.49), and the percent changes in FTV (12 vs 14 m; *p* = 0.49) and TLF (5.6 vs 17 m; *p* = 0.54) also were not significant.

**Conclusion:**

Higher TLF at baseline and higher SUVmax at follow-up NaF PET/CT corresponded with shorter survival in patients with bone metastases from urological malignancies who underwent treatment. NaF PET/CT may be a useful predictor of OS in this population.

## Introduction

Tyrosine kinase inhibitors (e.g., cabozantinib) and immune checkpoint inhibitors that block PD-1 (e.g., nivolumab) and CTLA-4 (e.g., ipilimumab) are promising treatments for patients with metastatic urological malignancies [1–4]. Previous studies using the combination of cabozantinib and nivolumab with or without ipilimumab have demonstrated encouraging antitumor activity [5].

With novel therapies whose mechanisms of action differ from conventional chemotherapeutic agents, predicting treatment response and patient prognosis is challenging [6]. Functional imaging with PET/CT can be useful in evaluating drug effects on tumor, and thereby in guiding clinical strategies to optimize treatment and minimize side effects.

Tumor burden parameters derived from whole body ^18^F-sodium fluoride (NaF) PET/CT have been used to predict overall survival (OS) in patients with prostate cancer and breast cancer metastatic to bone [7–9]. However, no study has used whole body bone fluoride volume from NaF PET/CT in patients with urological malignancies treated with combination therapy. The purpose of this study was to evaluate the prognostic value of NaF PET/CT in patients with urological malignancies treated with cabozantinib and nivolumab with or without ipilimumab.

## Materials and methods

### Patients

**Fig. 1:**
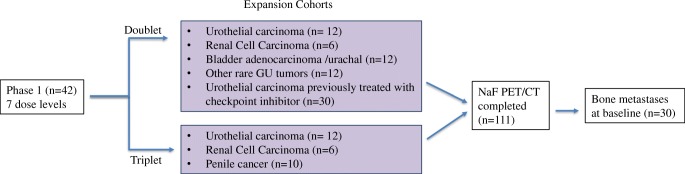
Schema of phase 1 and expansion cohorts of cabozantinib, nivolumab, and ipilimumab. Doublet, cabozantinib + nivolumab; triplet, cabozantinib + nivolumab + ipilimumab. Expansion cohorts are still accruing, NaF PET/CT no longer mandatory for protocol enrollment

This study was approved by the National Cancer Institute’s Institutional Review Board and conducted at the Center for Cancer Research in Bethesda, MD. Written informed consent was obtained from each patient enrolled in this phase I, 2-arm safety study of cabozantinib plus nivolumab alone (CaboNivo) or in combination with ipilimumab (CaboNivoIpi) in patients with metastatic genitourinary tumors. In this manuscript, we report on an exploratory endpoint of the study, which is to assess the prognostic value of NaF in patients with GU tumors and bone metastases treated with CaboNivo or CaboNivoIpi. Patients (*n* = 111) included 88 males and 23 females aged 20 to 82 years with histologically confirmed genitourinary tumors (Fig. 1). Thirty patients had bone metastases and an Eastern Cooperative Oncology Group (ECOG) performance status of 0–2, including patients with urothelial carcinoma (*n* = 15), prostate cancer (*n* = 5), renal medullary carcinoma (*n* = 3), mucinous adenocarcinoma (*n* = 2), penile carcinoma (*n* = 2), testicular carcinoma (*n* = 2), and primitive neuroectodermal tumor (*n* = 1).

### PET/CT imaging techniques

All patients underwent sequential ^18^F-FDG PET/CT and NaF PET/CT at baseline and 8 weeks post-treatment. One hour after injection with 185–370 MBq (5–10 mCi) of FDG, imaging was acquired on a Gemini TF PET/CT scanner (Philips Healthcare, Amsterdam, The Netherlands) from the base of the skull to the upper thigh. Immediately, after acquisition of the FDG PET/CT scan, patients were injected with 111–185 MBq (3–5 mCi) of NaF; 1 h after NaF injection, imaging was performed on the same PET/CT scanner from the vertex of the skull to the feet.

Low-dose CT transmission scans were obtained (120 kVp, 60 mAs, 0.75-s rotation time, 1.438 pitch, and 5-mm axial slice thickness) for attenuation correction and localization. Emission PET images were obtained at 2 min per bed position with 22 overlapping slices per bed. PET images were reconstructed using the Gemini TF default reconstruction algorithm: BLOB-OS-TF, a 3-dimensional ordered-subset iterative time-of-flight reconstruction technique using 3 iterations, 33 subsets, and 4 × 4 × 4 mm voxels. Imaging review and analysis were performed using a MIM workstation version 6.5.6 (MIM Software Inc., Cleveland, OH).

### Imaging analysis

NaF PET/CT scans with bone metastases (*n* = 49; 30 at baseline, 19 at 8 weeks) were analyzed by 3 nuclear medicine physicians (IL, MLL, EM). The first half of the NaF PET/CT scans were done together, during which analysis parameters were set. In subsequent analyses, differences between readers were resolved through consensus. Whole body bone fluoride volume as a secondary marker for bone metastases was measured on all NaF PET/CT scans using methods similar to those described by Etchebehere et al. [8].

**Fig. 2:**
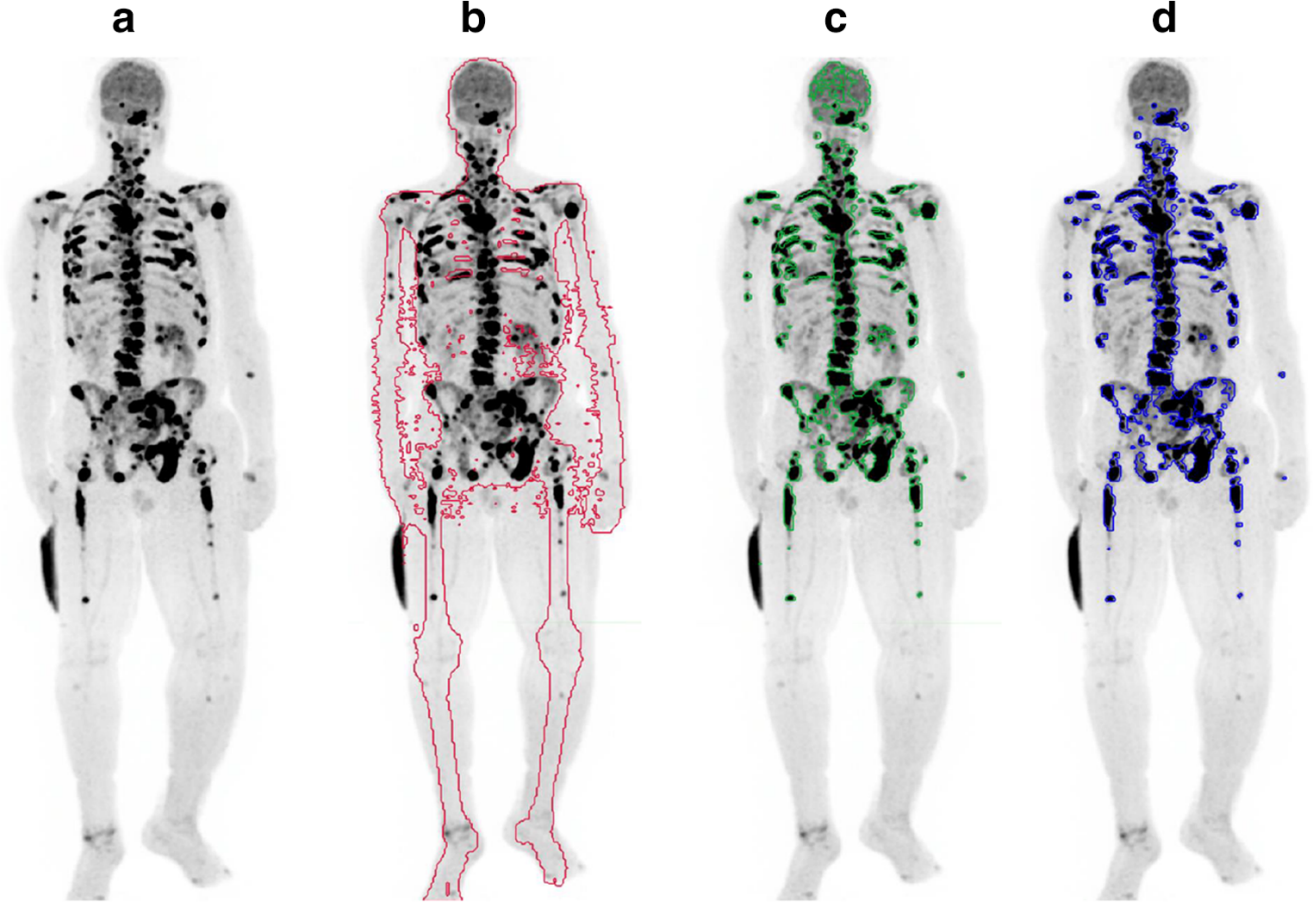
Quantification of SUVmax and whole body bone fluoride parameters (FTV and TLF) on NaF PET/CT. **a** NaF PET/CT of patient with urothelial carcinoma being treated with nivolumab 1 mg/kg, cabozantinib 40 mg, and ipilimumab 1 mg/kg. **b** Semi-automatic VOI is drawn in bone density contour. **c** With the threshold SUVmax of ≥ 10, all background activity is removed, leaving metastases and some non-metastatic VOIs with high uptake. **d** All non-metastatic VOIs (e.g., urine activity and brain activity) are removed. Quantitative analysis showed SUVmax of 137.1 (right pelvic bone), FTV of 719 ml, and TLF of 17,875. After 2.8 months of treatment, this patient died due to disease progression

Whole body bone density contours were automatically segmented on PET from the CT image through a MIM workflow. From this bone density contour, volumes of interest (VOIs) were automatically generated with a maximum standardized uptake value (SUVmax) threshold of 10. This VOI was then assessed manually for bone metastasis, and non-pathologic uptake such as urine activity, injection-site activity, and degenerative lesions was removed. The following parameters were then obtained per scan: highest SUVmax among all metastatic lesions (SUVmax), whole body fluoride bone tumor volume (FTV), and total lesion fluoride (TLF). TLF is calculated as SUVmean multiplied by FTV (Fig. 2). Percent change in SUVmax and FTV was calculated from scans obtained at baseline and 8 weeks post-treatment. The SUV activity of FDG did not influence NaF analysis because the highest FDG SUV activity did not surpass the threshold of SUV of 10 used for NaF PET.

### Statistical analysis

The Mann-Whitney test was used to compare between the CaboNivo and CaboNivoIpi groups. Individual prognostic factors (age, ECOG performance, lymph node metastasis, visceral metastasis, SUVmax1, SUVmax2, FTV1, FTV2, TLF1, TLF2, ΔSUVmax, ΔFTV, change of SUVmax, change of FTVtumor) were analyzed by univariate analysis using the Kaplan-Meier method and log-rank test. All statistical evaluations were performed using MedCalc software version 12.0.3.0 (MedCalc Software, Mariakerke, Belgium). A *p* value of < 0.05 was considered statistically significant.

## Results

### Patient characteristics

Starting in September 2015, 30 patients with bone metastases were treated with CaboNivo (*n* = 14) or CaboNivoIpi (*n* = 16). Four patients survived up to the observation period of October 2018. A total of 49 NaF PET/CT scans on 30 patients with urological malignancies were evaluated (30 at baseline, 19 at 8 weeks post-treatment, see Table 1 for patient details and tumor characteristics).

**Table 1:** Patient characteristics

	Total	CaboNivo	CaboNivoIpi
No. of patients	30	14	16
Median age (range)	55 (20–82)	54 (33–75)	57 (20–82)
Sex			
- Female	1	0	1
- Male	29	14	15
ECOG performance status			
0	1	1	0
1	21	10	11
2	8	3	5
Nodal metastases			
- No	6	4	2
- Yes	24	10	14
Visceral metastases			
- No	13	8	5
- Yes	17	6	11
Nivolumab			
- 1 mg/kg	12	5	7
- 3 mg/kg	18	9	9
Cabozantinib			
- 40 mg	23	8	15
- 60 mg	6	5	1
Ipilimumab			
- 1 mg/kg	14		14
- 3 mg/kg	2		2
NaF imaging no.			
- Baseline	30	14	16
- Follow-up	19	10	9
Histology			
- Mucinous adenocarcinoma	2	2	0
- P-NET	1	0	1
- Penile cancer	2	0	2
- Prostate cancer	5	4	1
- Renal medullary carcinoma	3	0	3
- Testicular cancer	2	2	0
- Urothelial carcinoma	15	6	9

*CaboNivo*, cabozantinib plus nivolumab alone; *CaboNivoIpi*, cabozantinib plus nivolumab combination with ipilimumab; *NaF*, sodium fluoride; *P-NET*, primitive neuroectodermal tumor

### Imaging parameters from NaF PET/CT

The SUVmax at baseline NaF PET/CT ranged from 16.7 to 157.7 (median 43). The SUVmax tendency toward decrease at follow-up NaF PET/CT ranged from 21.3 to 91.3 (median 31). The FTV at baseline NaF PET/CT ranged from 9.6 to 1570 ml (median 439 ml). The FTV at follow-up NaF PET/CT ranged from 56 to 6290 (median 448). SUVmax, FTV, and percent change showed no significant difference between the CaboNivo and the CaboNivoIpi groups at baseline or follow-up.

### Survival analysis

Thirty patients with bone metastases from urological malignancies were analyzed. Patient survival ranged 0.23–26 months (median 5.7 months), except for 4 patients who survived past the observation period. Prognostic factors of OS were assessed with the Kaplan-Meier survival method using diverse parameters. The median values of diverse parameters were used as dichotomization thresholds for Kaplan-Meier survival analysis.

**Fig. 3:**
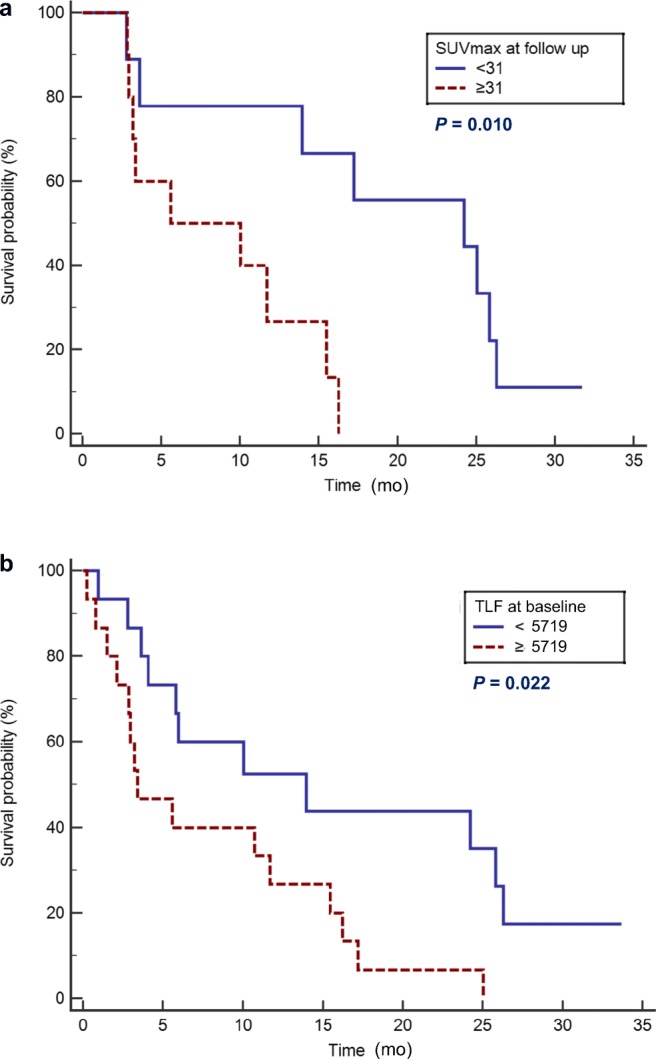
Kaplan-Meier analysis of overall survival according to TLF at baseline (**a**) and SUVmax at follow-up (**b**) using NaF PET/CT. Patients classified as higher TLF at baseline and higher SUVmax at follow-up had poorer OS. Log-rank test revealed significant differences in respective groups (*p* = 0.022 for TLF at baseline and *p* = 0.010 for SUVmax at follow-up)

A higher SUVmax at follow-up NaF PET/CT was found to correspond significantly with shorter OS (hazard ratio [HR] 4.81, 95% confidence interval [CI] 1.45–16.0, *p* = 0.010; Fig. 3a). A higher TLF at baseline NaF PET/CT was also found to correspond significantly with shorter OS (HR 2.64, 95% CI 1.15–6.06, *p* = 0.022; Fig. 3b). TLF at baseline NaF PET/CT showed the same results with FTV at baseline NaF PET/CT because FTV and TLF at baseline NaF PET/CT were highly correlated (rho = 0.97; *p* < 0.0001). Therefore, only TLF was analyzed and described afterwards. OS visceral metastasis was associated with a not statistically significantly shorter OS (*p* = 0.11). Age, ECOG performance status, nodal metastasis, baseline SUVmax, ΔSUVmax, ΔFTV, and change of SUVmax, FTV, or TLF were not significant factors in OS (Table 2).

**Table 2:** Survival analysis of ^18^F-sodium fluoride PET/CT parameters and clinical subgroups in genitourinary malignancy patients with bone metastases treated with cabozantinib, nivolumab, with or without ipilimumab

Variable	*N*	Median survival* (m)	*p* value
Age			0.25
< 56 years	15	5.6 [3.2 to 15.5]	
≥ 56 years	15	10.7 [4.1 to 25.8]	
ECOG			0.13
0, 1	22	10.7 [5.6 to 17.2]	
2	8	1.5 [0.8 to 11.7]	
Nodal metastasis			0.48
No	6	10.7 [2.8 to 25.0]	
Yes	24	5.8 [3.4 to 15.5]	
Visceral (non-bone) metastasis			0.11
No	13	16.2 [6.0 to 25.0]	
Yes	17	3.6 [2.1 to 13.9]	
NaF baseline SUVmax			0.85
< 43.1	15	5.8 [3.4 to 15.5]	
≥ 43.1	15	10.7 [3.2 to 17.2]	
NaF baseline FTV (ml)			0.022
< 439	15	13.9 [5.8 to 26.3]	
≥ 439	15	3.4 [2.8 to 11.7]	
NaF baseline TLF			0.022
< 5729	15	13.9 [5.8 to 26.3]	
≥ 5729	15	3.4 [2.8 to 11.7]	
NaF SUVmax 8 weeks post-treatment			0.010
< 31	9	24.2 [13.9 to 25.8]	
≥ 31	10	5.6 [3.2 to 15.5]	
NaF FTV (ml) 8 weeks post-treatment			0.49
< 448	9	17.2 [10.0 to 25.8]	
≥ 448	10	5.6 [2.9 to 16.2]	
NaF TLF 8 weeks post-treatment			0.49
< 5719	9	17.2 [10.0 to 25.8]	
≥ 5719	10	5.6 [2.9 to 16.2]	

Figures in brackets are 95% confidence intervals

*Univariate Kaplan-Meier analysis for overall survival

*ECOG*, Eastern Cooperative Oncology Group; *SUV*, standardized uptake value; *FTV*, total volume of fluoride avid bone metastases; *TLF*, total fluoride skeletal metastatic lesion uptake

## Discussion

This study demonstrates that bone metastasis FTV or TLF at baseline and SUVmax at 8-week follow-up on NaF PET/CT can predict OS after treatment with cabozantinib and nivolumab with or without ipilimumab in patients with urological malignancies. These results are consistent with those of earlier studies [8–10] in which skeletal tumor burden from NaF PET/CT predicted patient outcomes. One of these studies showed that, similar to our results, whole bone metastasis volume at baseline predicted OS in patients with prostate cancer [8]. However, that study acquired only baseline NaF PET/CT. We found that SUVmax at 8-week follow-up NaF PET/CT also correlated with OS. Previous studies using progression-free survival have shown similar results [9]. However, ours is the first study to demonstrate the usefulness of NaF PET/CT in patients with urological malignancies treated with cabozantinib plus nivolumab with or without ipilimumab. The results of this study confirm that we can classify patients earlier based on the bone metastasis uptake assessment before and shortly after treatment, enabling clinicians to optimize therapy strategies earlier in patients who are more likely to see benefit.

Since NaF does not directly target tumor but captures the osteoblastic response, the predictive value of fluoride bone volume on baseline NaF PET/CT likely parallels the burden of malignant cells activating bone turnover before treatment, which correlates to outcomes. A higher tumor load has lower therapy success and consequently a shorter survival time. In previous studies, fluoride bone volume determined by baseline NaF PET/CT was found to be a prognostic factor for OS and progression-free survival in prostate cancer and breast cancer patients [7, 8].

The predictive value of SUVmax at the 8-week follow-up NaF PET/CT could indirectly reflect tumor cell aggressiveness after treatment, as treatment stimulates osteoblastic activity. A previous study has also demonstrated the predictive role of SUVmax at the 8-week follow-up NaF PET/CT in progression-free survival (HR 1.61, 95% CI 1.14–2.28, *p* = 0.006) [9].

The pattern of treatment response with novel anticancer agents such as immune checkpoint inhibitors is different from conventional tumoricidal chemotherapeutic agents [11–13]. Some responders showed an early inflammatory response that is challenging to interpret on conventional anatomical imaging such as CT and MRI [14]. Immune-related response criteria have been helpful, but still have limitations [6]. Moreover, it is difficult to understand treatment response or prognosis after combination treatment with immune checkpoint inhibitors and a tyrosine kinase inhibitor. Functional molecular imaging with FDG PET/CT appears to be helpful in evaluating patients treated with immune checkpoint inhibitors [15–17]. The present study employed another functional imaging agent, NaF PET/CT, to successfully analyze whole body bone volume after combination treatment with immunotherapy and a tyrosine kinase inhibitor.

The quantitative PET parameters used in the present study are similar to those used in previous studies and are reproducible [8, 18]. Whole body bone fluoride volume can be obtained semi-automatically and thus can be operator independent. Using this quantitative method, we found that bone fluoride volume as a surrogate for bone metastasis predicts OS in patients with urological malignancies, just as it does in prostate cancer patients [8]. Furthermore, this form of imaging analysis can be used for prognosis in other cancers that metastasize to bone.

In NaF PET/CT, the possibility of flare phenomenon should be considered [19, 20]. In the present study, higher NaF SUVmax and TLF groups showed worse survival (Table 2). Therefore, we infer that an increase in SUV is more consistent with disease progression than with a flare phenomenon. Our study has several limitations. First, the patient population had heterogeneous tumors, although most of them had urothelial carcinoma. Second, there were only a limited number of patients with bone metastases. A large-scale study of patients with bone metastases is necessary to verify our results and to demonstrate the clinical significance of our findings. Third, 11/30 patients did not complete the 8-week follow-up NaF PET/CT scan due to progression of disease (*n* = 6), amendment of protocol (*n* = 2), sepsis (*n* = 1), dementia (*n* = 1), and different follow-up schedule (*n* = 1).

## Conclusion

NaF PET/CT can predict OS after treatment with cabozantinib and nivolumab with or without ipilimumab in patients with urological malignancies in terms of total lesion fluoride uptake at baseline NaF PET/CT and SUVmax at 8-week follow-up NaF PET/CT. Our study demonstrates that NaF PET/CT may be a useful prognostic tool for patients with urological malignancies metastatic to bone.
